# Associations between preweaning calf feeding behaviors with age at first calving and lactational performance using an automatic calf feeder

**DOI:** 10.3168/jdsc.2022-0255

**Published:** 2022-12-14

**Authors:** T.H. Swartz, C.S. Petersson-Wolfe

**Affiliations:** 1 Department of Animal Science, Michigan State University, East Lansing 48824; 2 Department of Dairy Science, Virginia Tech, Blacksburg 24061

## Abstract

The objective of this retrospective observational study was to determine whether preweaning calf behaviors and milk replacer (MR) intake from an automatic calf feeder were associated with age at first calving and first-lactation performance. Calves were housed in groups with access to an automatic calf feeder for 7 wk with a maximum milk allowance of 1,800 g/d of MR (12 L/d). Outcomes of interest included age at first calving (n = 137), first-lactation mature-equivalent 305-d (ME305) milk yield (n = 132), and first-lactation ME305 energy-corrected milk (ECM) yield (n = 132). Linear models included the fixed effects of the daily means of unrewarded visits, rewarded visits, drinking speed, and MR intake. Furthermore, breed, disease diagnosis, season of birth, and age of the calf when it was first introduced to the automatic calf feeder were included in all models. The genetic parameter for milk yield (predicted transmitting ability for milk) was included in models related to lactational performance. Feeding behaviors and milk replacer intake were not associated with age at first calving. Unrewarded visits to the automatic calf feeder were associated with ME305 milk and ECM yields. As mean daily unrewarded visits increased by 1, first-lactation ME305 milk yield and ME305 ECM yield increased by 319 kg and 224 kg, respectively. No other feeding behavior was significantly associated with first-lactation ME305 milk or ECM yields. In conclusion, unrewarded visits were positively associated with first-lactation performance, but external validation is still needed.

## Highlights

• Unrewarded visits were positively associated with first-lactation ME305 milk yield.
• Unrewarded visits were positively associated with first-lactation ME305 energy-corrected milk yield.
• Preweaning calf feeding behaviors may be associated with long-term performance.

## Graphical Abstract

**Figure:**
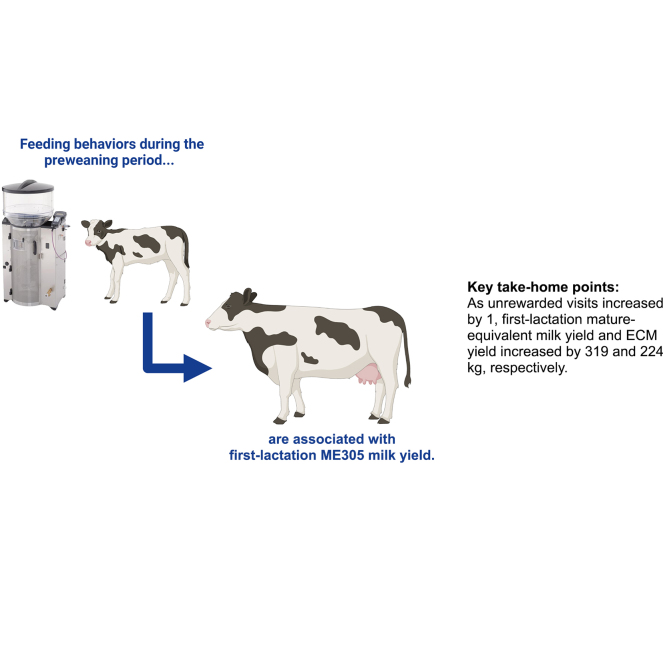
**Summary:** The goal of this study was to determine whether preweaning calf behaviors recorded by an automatic calf feeder were associated with performance in adulthood. As the number of unrewarded visits to the feeder increased, first-lactation mature-equivalent milk yield and energy-corrected milk yield increased. These associations justify additional investigation into the use of preweaning calf feeding behaviors to predict firstlactation milk yield.

Automated calf feeders have begun to gain popularity in the dairy industry. These feeders have generally been used to facilitate the feeding of high milk allowances to calves without requiring additional labor to do so (Kung et al., 1997). The automatic calf feeder records milk intakes and feeding behaviors. These feeding behaviors include unrewarded visits (non-nutritive visit), rewarded visits (calf receives milk), and drinking speed (mL/min). Currently, automatic calf feeder data may be used for identifying diseased calves [neonatal calf diarrhea (**NCD**) and bovine respiratory disease (**BRD**)]. Diseased calves have fewer unrewarded visits (Svensson and Jensen, 2007; Knauer et al., 2017), fewer rewarded visits (Conboy et al., 2022), reduced drinking speeds (Knauer et al., 2017), and they consume less milk (Knauer et al., 2017; Swartz et al., 2017; Cantor and Costa, 2022; Conboy et al., 2022) than healthy calves, although it should be noted that some of these associations are dependent on milk allowance (Borderas et al., 2009) and the disease diagnosed (Knauer et al., 2017).

Another potential use for these data could be predicting long-term performance. Indeed, some studies (Soberon et al., 2012; Soberon and Van Amburgh, 2013) but not all (Morrison et al., 2009; Terré et al., 2009; Chester-Jones et al., 2017; Korst et al., 2017) have found that calves that had greater milk replacer (**MR**) intakes produced more milk during their first lactation. Furthermore, preweaning ADG was positively associated with first-lactation milk yield (Gelsinger et al., 2016). Moreover, calves that became ill during the preweaning period produced less first-lactation milk yield (Heinrichs and Heinrichs, 2011; Abuelo et al., 2021). Additionally, behaviors learned during the preweaning period may persist after weaning, and therefore could impact long-term performance (Miller-Cushon and DeVries, 2015). For instance, preweaning calves fed greater milk intake levels (ad libitum vs. 5 L/d) consumed solid feed more slowly and in smaller meals during the postweaning period, although these effects dissipated as the calf became older (Miller-Cushon et al., 2013). Nevertheless, it is still unknown if the behaviors recorded by the automatic calf feeder are associated with long-term performance. It is plausible that behavioral tendencies found during the preweaning period may be associated with behaviors found in adulthood (Miller-Cushon and DeVries, 2015) that are also associated with lactational performance. Furthermore, feeding behaviors are associated with health status, and disease during the preweaning period is associated with poorer performance in adulthood (Heinrichs and Heinrichs, 2011; Abuelo et al., 2021). Therefore, the objective of this study was to determine whether preweaning calf feeding behaviors (unrewarded visits, rewarded visits, and drinking speed) and MR intake from the automatic calf feeder were associated with age at first calving and first-lactation milk yield and ECM yield. We hypothesized that MR intake, the number of unrewarded and rewarded visits, and drinking speed would be negatively associated with age at first calving and positively associated with milk yield.

Ethical approval was not needed as the study was an observational study and only routine animal husbandry procedures were performed. Preweaning calf data from November 2014 through August 2016 were used in this retrospective observational study from the Virginia Tech dairy (Blacksburg, VA). The farm protocol for managing newborn calves was as follows: The calf was removed from the dam as soon as the calf was found, and navels were dipped with 7% iodine. Holstein calves were fed 4 L of colostrum, and Jersey calves were fed 3 L as soon as possible following birth. A second feeding of colostrum of similar quantity and quality was provided approximately 12 h following the first feeding. If a calf did not consume colostrum, an esophageal tube was used. Colostrum quality was assessed using a Brix refractometer. Fresh colostrum was fed if the Brix score was ≥23%; otherwise, frozen colostrum (with a Brix score ≥23%) or colostrum replacer was fed. Calves received an intranasal vaccine (Inforce 3, Zoetis) on the day of birth.

During this observational study, 206 female calves were born. Of these, 146 calves (104 Holstein and 42 Jersey calves) reached maturity and reproduced for the first time (i.e., reached first calving). All calves were housed in individual hutches until they were moved into group-housing (median age, 7 d; mean, 7.8 d; SD, 1.9 d; interquartile range, 1.0 d). While in hutches, calves were fed 2 L of approximately 41°C water mixed with 300 g of MR twice daily. Calves were fed a 26% CP and 20% crude fat milk replacer (Cow's Match Cold Front, Land O'Lakes Animal Milk Products Co.) during the winter to offset increased energy requirements due to cold stress. Otherwise, calves were fed a 27% CP and 10% crude fat milk replacer (Cow's Match Warm Front, Land O'Lakes Animal Milk Products Co.). The group-housing pen had a sawdust bedded pack that was maintained by the farm staff. Because calves were continuously added to the group and left the pen following weaning, group size was dynamic, with approximately 10 calves in the group at any given time. While in group housing, calves were allowed ad libitum access to an automatic waterer and calf starter (Intensity 22% Textured Calf Starter Medicated, Southern States) provided in a trough. Calves were fed using an automatic calf feeder (FA Förster-Technik GmbH) for 7 wk, starting from the day each calf entered group housing. The automatic calf feeder gradually increased milk allowances from 900 g/d (6 L/d) to 1,800 g/d (12 L/d). Maximum milk allowance was reached 10 d after calves were placed onto the feeder. Weaning began 10 d before the final day on the feeder; the milk allowance was gradually reduced until calves were weaned after 7 wk (on d 49). We define the preweaning period to be the period when the calf is receiving any milk, including the periods where milk allowances were variable (ramp-up and weaning periods) and stable. The automatic calf feeder recorded unrewarded visits (number of visits to the feeder without a milk entitlement), rewarded visits (number of visits to the feeder with a milk entitlement), drinking speed (mL/min), and MR intake (g). Unrewarded and rewarded visits were recorded daily, totaled over the preweaning period, and then expressed as a daily average to provide a single value for each calf. Drinking speed and MR intake were recorded at each visit. Drinking speed was averaged across visits per day and then averaged across all days during the entire preweaning period to provide a single value for each calf. Milk replacer intake was summed across visits by day and then averaged across all days during the entire preweaning period to provide a single daily value for each calf.

Calves were weighed at birth using a digital scale by the farm staff, and these data (83 birthweights were available; 63 were missing) were entered into herd management software (PCDart, Dairy Records Management Services). Lactation records and age at first calving data were also obtained from the herd management software. Milk composition and yield were determined for individual cows at approximately monthly intervals by DHIA technicians. Energy-corrected milk yield was determined using the following equation: (0.327 × milk yield) + (12.95 × milk fat yield) + (7.65 × milk protein yield). Mature-equivalent (**ME305**) 305-d lactation yields were calculated by DHIA for animals that remained in the herd for at least 90 d for each lactation (5 first-lactation animals were culled before 90 d). Records from heifers that never calved were not available.

Health records were obtained from herd management software (PCDart, Dairy Records Management Services) for calves during the preweaning period that were treated for NCD (n = 44), BRD (n = 34), both NCD and BRD (n = 7), or not diagnosed with any calfhood disease (n = 61). Diseased calves were diagnosed by herd veterinarians or by the farm staff (who were trained by the herd veterinarians). The herd veterinarian typically visited the farm multiple times a week, and calves were visually observed daily by the farm staff. Neonatal calf diarrhea was diagnosed when the feces were loose (substantial spreading) or watery (sifts through the bedding); BRD was diagnosed when calves had any combination of elevated body temperature, nasal discharge, eye discharge, ear tilt, or a cough. Disease interventions were determined by the farm staff under advisement from the herd veterinarian. Calves treated for NCD received electrolytes, a nonsteroidal anti-inflammatory drug (Banamine, Merck Animal Health), and an injectable antibiotic (Excenel RTU, Zoetis Inc.). Calves diagnosed with BRD were treated with an injectable antibiotic (either Draxxin, Zoetis Animal Health, or Resflor Gold, Merck Animal Health).

Some calves were removed from the automatic feeder before completing the 7-wk feeding program and were therefore excluded from the analyses (n = 9). As a result, the final sample size was 137 for age at first calving. Because 5 first-lactation animals were culled before 90 DIM, the final sample size for first-lactation ME305 milk and ECM yields was 132. No other exclusion criteria were used.

Preweaning calf behaviors (unrewarded visits, rewarded visits, and drinking speed) and MR intake were considered for model inclusion. To determine whether behaviors and MR intake were correlated, associations between these variables were assessed using Pearson correlations (PROC CORR, SAS Institute Inc.). Linear models (PROC GLIMMIX, SAS Institute Inc.) were used to evaluate the associations of the calf's preweaning feeding behaviors and MR intakes with age at first calving and first-lactation ME305 milk and ECM yields. Fixed effects included the linear terms for the daily means of unrewarded visits, rewarded visits, drinking speed, and MR intake recorded by the automatic calf feeder during the preweaning period. In addition to those, the calf's age at introduction to the automatic calf feeder (linear term), breed (Holstein versus Jersey), the season the calf was born (winter, summer, fall, or spring), and disease diagnosis during the preweaning period (NCD, BRD, both NCD and BRD, or healthy) were included in the model. For first-lactation ME305 milk and ECM yields, the genetic parameter for milk yield (PTA for milk; **PTAM**) was also included in the model. Birth weight (linear term) was tested in all models; however, it was never significant and therefore removed from the analyses. Residuals were assessed for normality and outliers (PROC UNIVARIATE, SAS Institute Inc.); no outliers were identified.

Descriptive statistics are provided in Table 1. No strong correlations were found between mean daily MR intake, drinking speed, rewarded visits, and unrewarded visits (all |r| <0.7; Table 2).

**Table 1:** Descriptive statistics for preweaning calf feeding behaviors, milk replacer intake, and long-term performance, including age at first calving (n = 137) and first-lactation performance (n = 132)^1^

Item	Mean	SD	IQR^2^
Mean daily unrewarded visits (no./d)	3.8	2.1	2.1
Mean daily rewarded visits (no./d)	7.2	2.3	2.1
Mean daily visit drinking speed (mL/min)	587	129	156
Mean daily milk replacer intake (g)	1,054	148	215
Age at first calving (d)	706	47	60
First-lactation performance			
ME305^3^ milk yield (kg)	13,659	2,413	3,822
ME305 protein yield (kg)	438	62	86
ME305 fat yield (kg)	519	68	103
ME305 ECM yield (kg)	14,539	1,926	2,934

1 Calves were placed on the automatic calf feeder at 7.8 ± 1.9 d of age (mean ± SD). The automatic calf feeder gradually increased milk allowances from 900 g/d (6 L/d) to 1,800 g/d (12 L/d). Maximum milk allowance was reached 10 d after calves were placed onto the feeder. Weaning began 10 d before the final day on the feeder where the milk allowance was gradually reduced until calves were weaned after 7 wk (on d 49).

2 IQR = interquartile range.

3 ME305 = mature-equivalent.

**Table 2:** Pearson correlation coefficients among preweaning calf behaviors (unrewarded visits, rewarded visits, and drinking speed) and milk replacer (MR) intake recorded by the automatic calf feeder during the preweaning period^1^

Variable	Unrewarded visits (no./d)	Rewarded visits (no./d)	Drinking speed (mL/min)	MR intake (g)
Unrewarded visits (no./d)	—	0.10	0.37	0.51
Rewarded visits (no./d)	0.10	—	−0.06	0.06
Drinking speed (mL/min)	0.37	−0.06	—	0.42
Milk replacer intake (g)	0.51	0.06	0.42	—

1 Calves were placed on the automatic calf feeder at 7.8 ± 1.9 d of age (mean ± SD). The automatic calf feeder gradually increased milk allowances from 900 g/d (6 L/d) to 1,800 g/d (12 L/d). Maximum milk allowance was reached 10 d after calves were placed onto the feeder. Weaning began 10 d before the final day on the feeder where the milk allowance was gradually reduced until calves were weaned after 7 wk (on d 49).

Feeding behaviors [unrewarded visits (*P* = 0.91), rewarded visits (*P* = 0.93), and drinking speed (*P* = 0.63)] and MR intake (*P* = 0.20) were not significantly associated with age at first calving. Breed (*P* = 0.97), birth season (*P* = 0.99), and disease diagnosis (*P* = 0.61) were not associated with age at first calving; however, age of calf at introduction to the automatic calf feeder was associated with age at first calving (*P* = 0.03; Table 3). As the age of calf at introduction to the automatic calf feeder increased by 1 d, age at first calving increased by 5.2 d.

**Table 3:** Multivariate analysis to assess the associations of preweaning calf behaviors and milk replacer intake with age at first calving (n = 137) and mature-equivalent 305-d first-lactation milk and ECM yields (n = 132)

Variable	Estimate	SE	*P*-value	95% CI
Age at first calving (d)				
Mean daily unrewarded visits	−0.28	2.42	0.91	−5.1, 4.5
Mean daily rewarded visits	0.18	2.12	0.93	−4.0, 4.4
Mean daily visit drinking speed (mL/min)	−0.018	0.038	0.63	−0.093, 0.057
Daily milk replacer intake (kg)	0.045	0.035	0.20	−0.11, 0.024
Age at introduction to the automatic calf feeder (d)	5.2	2.3	0.03	0.62, 9.73
Breed			0.97	
Birth season			0.99	
Disease diagnosis			0.61	
Intercept	727	37	<0.0001	654, 800
Milk yield (kg)				
Mean daily unrewarded visits	319	84	<0.001	153, 485
Mean daily rewarded visits	−104	72	0.15	−248, 39
Mean daily visit drinking speed (mL/min)	−2.3	1.3	0.08	−4.9, 0.28
Daily milk replacer intake (kg)	−2.1	1.2	0.10	−4.50, 0.39
Age at introduction to the automatic calf feeder (d)	−13	79	0.87	−169, 144
PTA for milk (kg)	3.71	0.56	<0.0001	2.60, 4.81
Breed				
Holstein	3,315	358	<0.0001	2,806, 4,223
Jersey	Referent			
Birth season			0.40	
Disease diagnosis			0.96	
Intercept	13,644	1,283	<0.0001	11,102, 16,185
ECM yield (kg)				
Mean daily unrewarded visits	224	84	<0.01	57, 390
Mean daily rewarded visits	−125	73	0.09	−268, 19
Mean daily visit drinking speed (mL/min)	−1.6	1.3	0.24	−4.1, 1.1
Daily milk replacer intake (g)	−1.2	1.2	0.35	−3.6, 1.3
Age at introduction to the automatic calf feeder (d)	−41	79	0.61	−198, 116
PTA for milk (kg)	1.8	0.56	<0.01	0.69, 2.91
Breed				
Holstein	2,048	360	<0.0001	1,336, 2,761
Jersey	Referent			
Birth season			0.40	
Disease diagnosis			0.94	
Intercept	15,337	1,290	<0.0001	12,783, 17,891

Mean daily unrewarded visits to the automatic calf feeder were positively associated with first-lactation ME305 milk yield (*P* < 0.001; Table 3). As mean daily unrewarded visits increased by 1, first-lactation ME305 milk yield increased by 319 kg. Mean daily rewarded visits (*P* = 0.15), mean daily visit drinking speed (*P* = 0.08), and mean daily MR intake (*P* = 0.10) were not significantly associated with first-lactation ME305 milk yield. As expected, PTAM was positively associated with first-lactation ME305 milk yield (*P* < 0.0001), and Holstein cows had a greater first-lactation ME305 milk yield than Jersey cows (*P* < 0.0001). Birth season (*P* = 0.99), disease diagnosis (*P* = 0.61), and age of the calf at introduction to the automatic calf feeder (*P* = 0.87) were not associated with first-lactation ME305 milk yield.

Similarly, mean daily unrewarded visits to the automatic calf feeder were positively associated with first-lactation ME305 ECM yield (*P* < 0.01; Table 3). As mean daily unrewarded visits increased by 1, first-lactation ME305 ECM yield increased by 224 kg. Mean daily rewarded visits (*P* = 0.09), mean daily visit drinking speed (*P* = 0.24), and mean daily MR intake (*P* = 0.35) were not significantly associated with first-lactation ME305 ECM yield. Unsurprisingly, PTAM was positively associated with first-lactation ME305 ECM yield (*P* < 0.01), and Holstein cows had a greater ME305 ECM yield than Jersey cows (*P* < 0.0001). Birth season (*P* = 0.40), disease diagnosis (*P* = 0.94), and age of the calf at introduction to the automatic calf feeder (*P* = 0.61) were not associated with first-lactation ME305 ECM yield.

Heifer rearing is a costly investment that is of critical importance to optimize long-term performance (Van Eetvelde and Opsomer, 2017). As such, our objective was to assess associations of preweaning calf behaviors and MR intake with age at first calving and first-lactation performance. Although feeding behaviors and MR intake were not associated with age at first calving, unrewarded visits were positively associated with first-lactation performance. As such, data from the automatic calf feeder may have value extending beyond the preweaning period and could be useful for the selection of replacement animals.

Unrewarded visits were positively associated with ME milk and ECM yields. It should be noted that unrewarded visits were also moderately correlated with MR intake in the present study. Calves with more unrewarded visits are likely seeking more milk and have greater appetites. Indeed, a past study demonstrated that calves on a low milk allowance (approximately 4 L/d) had a greater number of unrewarded visits than calves on a high milk allowance (7 to 10 L/d; Jensen, 2009), indicating hunger (Jensen, 2006; De Paula Vieira et al., 2008). However, MR intake was not associated with ME305 milk or ECM yield in the present study, similar to some past studies (Morrison et al., 2009; Terré et al., 2009; Chester-Jones et al., 2017; Korst et al., 2017), but not all studies (Soberon et al., 2012; Soberon and Van Amburgh, 2013). Notably, preweaning ADG is positively associated with first-lactation milk yield (Soberon et al., 2012; Gelsinger et al., 2016). Similarly, grain intake during the preweaning period has been positively associated with first-lactation milk yield (Heinrichs and Heinrichs, 2011; Gelsinger et al., 2016). Individual grain intake was not measured in the present study. As such, future studies should consider this variable when assessing associations between preweaning feeding behaviors and intakes with long-term performance, as past studies have suggested a relationship. Nevertheless, we speculate that calves with more unrewarded visits to the automatic calf feeder may also have consumed greater amounts of grain to lessen their hunger. Potentially, calves that consume greater grain intakes during the preweaning period may also consume greater feed intakes in adulthood, subsequently enhancing milk yield. Future research should explore these behavioral tendencies further because tendencies found during the preweaning period may predict behaviors found in adulthood (Miller-Cushon and DeVries, 2015).

Another reason behind the positive association of unrewarded visits with milk yield could be related to disease. Past studies have associated a decrease in unrewarded visits in calves with neonatal calf diseases (Svensson and Jensen, 2007; Knauer et al., 2017). Continuing along those lines, calves that became ill during the preweaning period produce less first-lactation milk (Heinrichs and Heinrichs, 2011; Abuelo et al., 2021). As such, it is possible that the positive association of unrewarded visits with ME305 milk and ME305 ECM yield could be attributed to calfhood health. Recent work has highlighted the difficulty in diagnosing subclinical BRD (Ollivett and Buczinski, 2016), although it should be noted that subclinical BRD has not been associated with alterations in feeding behaviors (Cramer et al., 2020). Nevertheless, a potential limitation to our study is that the disease diagnosis was based on clinical signs and, as a result, calves with subclinical BRD would have gone unidentified.

Age of the calf at introduction to the automatic calf feeder was included in the model to control for variation and was not a direct objective of the present study. Nevertheless, we found an interesting association between this variable and age at first calving. More specifically, as the age of the calf at introduction to the automatic calf feeder increased by 1 d, age at first calving increased by 5.2 d. In the present study, placing the calf on the automatic feeder at a younger age would provide the calf with access to substantially greater MR allowances, especially because calves while housed in individual hutches were fed a minimal diet (600 g/d of MR). In agreement with this finding, past studies have found greater BW at puberty when feeding an intensive milk diet during the preweaning period (Davis Rincker et al., 2011). Therefore, calves that have access to greater amounts of MR at a younger age (due to an earlier introduction to the automatic feeder) may have grown faster and reached puberty sooner, subsequently reducing the age at first calving.

In conclusion, results from this retrospective observational study revealed that unrewarded visits recorded by the automatic calf feeder were positively associated with first-lactation performance. As such, future studies should assess whether unrewarded visits could be used to predict lactational performance in replacement animals, as the present study assessed associations and did not examine the predictive value of these data. A limitation to our study is that we assessed mean feeding behaviors over the entire preweaning period. Because feeding behaviors likely change during the different phases of the feeding program [i.e., when milk allowances are either increasing (ramp-up phase), stable, or decreasing (weaning phase) on the automatic calf feeder], it may be useful to assess associations of preweaning feeding behaviors and milk replacer intakes with long-term performance by each phase. Last, further investigation is required because results may depend on a variety of factors, including but not limited to milk allowance, feeding program and settings on the automatic calf feeder, disease prevalence, and preweaning calf group sizes.
